# Impact of social pediatrics rotation on residents’ understanding of social determinants of health

**DOI:** 10.1080/10872981.2022.2057791

**Published:** 2022-03-30

**Authors:** Kimberly Connors, Marghalara Rashid, Mercedes Chan, Jennifer Walton, Bonnieca Islam

**Affiliations:** a Department of Pediatrics, Faculty of Medicine and Dentistry, University of Alberta, Edmonton, Alberta, Canada; b Department of Pediatrics, University of British Columbia, Vancouver, BC, Canada

**Keywords:** Residency, social determinants of health, pediatrics, written reflection, grounded theory

## Abstract

Social Pediatrics is the newest mandatory rotation in the General Pediatrics residency program at the University of Alberta. Evaluation of the residents include a written reflective assignment, asking them to identify assets and disparities that have influenced the health of a child encountered on the rotation. While there are many published papers on reflective writing , few papers are found in the area of how social determinants of health (SDoH) impact an individual’s overall health. This study examines the question: how has exploring SDoH during the Social Pediatrics rotation led to changes in residents’ awareness of their own practice of pediatrics? Grounded theory was used to analyse 35 reflections from residents who had submitted them as a mandatory assignment at the end of their rotation. In addition, 10 semi-structured telephone interviews were conducted to further understand residents’ perceptions. Interviews were transcribed verbatim and analysis of the reflections and interviews was guided by grounded theory using open, axial, and selective coding. Analyses of written reflections revealed the following categories: 1) judgment/bias, 2) systemic challenges, 3) advocacy, and 4) a sense that everyone is doing their best. Interview data reinforced overlapping categories of bias, systemic challenges and advocacy in addition to two new categories: 1) increased exposure and knowledge of specific disadvantaged populations, and 2) understanding impact of SDoH on overall health.

Categories that were generated highlight the importance of residents’ education regarding the role of SDoH on overall health and management plans. They became aware of structural determinants of health working with health-care professionals who were advocates for the communities they worked with. Analysis of residents’ written reflection assignments and follow-up interviews revealed the value of reflective practice in physician development and reinforced the benefit of fostering experiences not typically encountered in traditional clinical learning environments.

## Introduction

Social determinants of health (SDoH) have been shown to have a greater impact on one’s overall health than one’s genetics or biology [1]. Yet, it is a topic that until recently, has not been recognized by medical schools as an important concept to include in medical school curricula. In the paper by Denizard-Thompson et al., they comment that ‘an increasing number of US medical schools have begun to recognize the need for health equity curricula that include issues such as access to care, housing instability, and racial/ethnic bias’ [2, pp.2/11]. They also reference papers that recognize that classroom-based experiences without community involvement may not be very beneficial [3]. Gilles Julien, who is considered to be the founder of Social Pediatrics in Canada, describes Social Pediatrics as a model of integrated social medicine [4]. He frames it as a community-based model which respects family cultures and the fundamental rights of children. It combines medicine, law and social sciences to identify needs and take effective action to reduce or eliminate toxic stressors that affect the development of children.

The Royal College of Physicians and Surgeons espouses that physician should function as health advocates and ‘responsibly use their expertise and influence to advance the health and well-being of individual patients, communities, and populations’ [5, pp.11]. A study done at the University of Ottawa medical school had a group of students during the Covid-19 pandemic, follow a social pediatric model for six weeks during clerkship. The control group completed the regular six week core pediatrics rotation. The intervention group had ‘a stronger overall grasp on the CanMEDS roles as compared to the control group. Such roles included those of Communicator, Health Advocate, Medical Expert, Collaborator, Leader/Manager and Professional’ [6, pp.2]. Pediatrics is the newest mandatory rotation added to the General Pediatric residency training program at the University of Alberta. Understanding the social, structural and political-economic determinants of health in the community (poverty, unemployment, food insecurity, early child development, health services, colonialism, etc.) is a necessary foundation to effectively provide recommendations and treatment to individual patients and their families. This foundation also provides residents with a framework and the skills necessary for intervention and advocacy to improve the health of children, families, and communities.

Many Canadian pediatric residency training programs now have a Social Pediatrics rotation, as a mandatory or an elective rotation. Other programs aim to have the objectives met by incorporating these concepts throughout other rotations. Our mandatory four-week rotation has been designed to provide focused training on community engagement, SDoH, advocacy, public health, health policy, and social justice. The rotation provides a community service learning opportunity for pediatric residents to gain a better appreciation of the environment in which their patients live, learn and grow; and how the environment affects patient health and accessibility to health services. The Social Pediatrics rotation aims to place residents in clinics or community programs that will give them exposure to the following seven populations that have unique needs and barriers to care: 1) adolescents, 2) inner city youth, 3) children in care, 4) child protection services, 5) indigenous children, 6) rural communities, and 7) new Canadians. Unlike other rotations in the residency program, the evaluation process involves a final written reflective assignment asking the resident to map and assess the assets and disparities that have influenced the health and life of a child they encountered on the rotation.

Reflective writing is becoming more common in medical school curricula, especially in North America. Chen and Forbes’ [7] review showed that reflective writing in medical education leads to many positive effects, including an increase in empathy towards patients. Another study examined an online curriculum on care delivery in the medical home. This was offered to third-year pediatric residents who were asked reflective questions [7]. The residents identified their need to improve their understanding of SDoH and systems issues to allow for better care for their patients and families. R.B. Levine et al. [8] looked at written reflections by internal medicine residents, who revealed the process led to deeper reflection and a desire to improve.

A paper by Plant et al. examined reflection in the clinical setting by pediatric residents and faculty. They found that although clinical reflection occurred, ‘they did not always explicitly identify it as reflection or reflect in growth-promoting ways’ [9, pp. S75]. A study by van den Heuvel et al. evaluated a social pediatrics elective for medical students through reflective writing. The most common theme that emerged was learners commenting on social determinants of health. The authors concluded transformative learning occurred through students’ new experiences with interacting with patients in their home communities and environments [10]. Although there are published papers on written reflections during medical school and some during residency training, limited research exists in the area of Social Pediatrics and the interplay between the social determinants of health (SDoH) and their impact on a patient’s ability to access care and the health-care team providing that care. Intentionally reflecting on the SDoH and their impact on a child’s health may increase health-care practitioners’ awareness of factors that influence their practice. The aim of our study was to collate and analyze general pediatrics residents’ written reflective assignments from a Social Pediatrics rotation to describe how exploring SDoH impacts resident’s awareness of their own practice.

## Methods

### Study design

11] was used for this study. In this qualitative design, the researcher generates a theory or uncover relationships and behavior as well as studies social processes. Ontologically grounded theory is rooted in sociological theories, such as pragmatism and symbolic interactionism. Grounded theory assumes that the objective reality is complex, overlapping, contradictory and that knowledge is developed retrospectively. Additionally, grounded theory also examines interactions and actions, and that the reality is intersubjective based on interactions and shared meanings. Epistemologically, it is rooted in positivism and constructionism. We have followed the positivist approach of Strauss and Corbin (1998), which states that objective reality should not be impacted by the researchers’ interpretations of it [11]. This means that we analysed our data objectively by making sure that the researches’ biases,feelings, and thoughts did not weave into the data. In this approach the phenomenon under investigation is ‘grounded’ in the databy using various sources and methods of data generation from study participants. Grounded theory approach was used as it enabled us to gain in-depth knowledge of how SDoH impacts postgraduate trainees while providing care to patients.

#### Study participants

Study participants were residents in an accredited Canadian Pediatrics Residency Program between 2016 and 2020 who had completed a 4 week Social Pediatrics rotation during their second year of training. This rotation was self-scheduled by the residents who were given a list of possible placements they may participate in, a diverse range of clinical experiences they are not routinely exposed to or made aware of during other rotations. One of our authors is the preceptor overseeing the rotation making sure schedules and objectives are reviewed. The resident also discusses their final written assignment with their preceptor one or two days after it has been submitted. In addition, following initial data analysis of reflection assignments (n = 35) and using purposeful sampling, a subset of these residents participated in follow-up telephone interviews (n = 10). This study was approved by the Health Research Ethics Board (HREB) at the University of Alberta.

### Data sources

The primary source of data for the grounded theory analysis were the written reflective assignments submitted by the residents. For these assignments, residents were required to identify a patient who had a significant impact on them during their rotation and, using the SDoH framework, describe and reflect upon both disparities and individual and community assets that influenced the health of that child. Residents also submitted disparities and assets maps as a visual aid to illustrate connections between individuals, their communities and their disparities and assets. In addition, residents were asked to reflect upon the following questions:

1) Were any of your assumptions challenged or validated?

2) Did you gain any significant insights about yourself (cognitive and/or emotional)?

3) How might your experience change your practice of medicine?

Following initial analysis of the written assignments, semi-structured telephone interviews lasting 35–60 minutes were conducted with a subset of study participants. All residents who had taken the course were invited to take part in the interviews. It is vital to note that we stopped collecting data because we had reached saturation. A point where we were not hearing any new information from our study participants. We developed an interview guide that was piloted with a resident on this study who had taken the Social Pediatrics rotation in previous years. Residents were asked to recall their overall experience with the Social Pediatrics rotation, elaborate on the patient interaction discussed in their written reflection and, if applicable, further discuss themes identified in their written reflection. These were performed by a resident (KC) and a qualitative researcher (MR). The first three interviews were done together for training purposes, and subsequent interviews were completed by KC.

### Data analysis

Three clinicians (KC, MC, JW) and one qualitative expert (MR) independently read the residents’ reflections. Finally, the principal investigator (BI), who is the Social Pediatrics rotation coordinator, also read and coded the data to confirm the identified categories and to ensure relevant information was not missed.

For our telephone interviews, two clinicians (KC, BI) and one qualitative expert (MR) read and analyzed the data. An iterative process of reading and coding each writing assignment and interview transcripts was used until categories reached saturation. Our analysis was guided by grounded theory and by employing the following three steps: *1) Open Coding*: Reflections and interviews were analyzed by describing common instances found throughout the data. In this phase, we were involved in breaking our data into segments that reflected particular meanings. *2) Axial Coding*: Once we were able to get familiar with our data and got a better understanding of which types of common codes were relevant to students in their social rotations, we then started labeling and assembling these codes into broad categories through constant comparison. *3) Selective Coding*: In this phase of our analysis, our categories were integrated and refined into larger abstract schema. During selective coding, decisions were made regarding data saturation. Saturation is a point at which during data analysis no new information appears to be arising from the data being analysed [12]. Saturation of categories was achieved through a search for repeated instances of categories, increased elaboration of identified categories, ongoing data review, and subsequent data collection, as needed.

### Trustworthiness

One of the first steps to ensure trustworthiness is by determining methodological congruence. This means evaluating the fit between the various components of the study design. For example, does the method fit with the research question? Does the type of strategy for data generation fit with the chosen method? Finally, is the analysis congruent with the strategy [13]. Furthermore, we used memos as one way to demonstrate trustworthiness by showing evidence of thoughts and creating an audit trail for study transferability. Throughout our study, we focused on developing appropriate codes that fit the data and the reflexive process of cross-checking new codes consistently to ensure reliability. To help minimize the effects of researchers’ presumptions, informal meetings (peer debriefing) were regularly conducted to discuss our thoughts, feelings and emotions while analyzing the data. By having regular meetings, it allowed us to step back from the data and determine whether we were bringing our own preferences into the analysis. We used memos during the analysis to keep track of decisions made in regard to coding and abstraction [13].

## Results

We analyzed 35 written reflections from second-year pediatrics residents who completed their Social Pediatrics rotation as part of their General Pediatrics residency at the University of Alberta. From those residents, 10 were recruited via theoretical sampling for a subsequent telephone interview. Of those, the majority remained in residency at the time of interview (PGY3 – 1, PGY4- 6, PGY5- 1) and one was a staff pediatrician.

**Figure 1.**
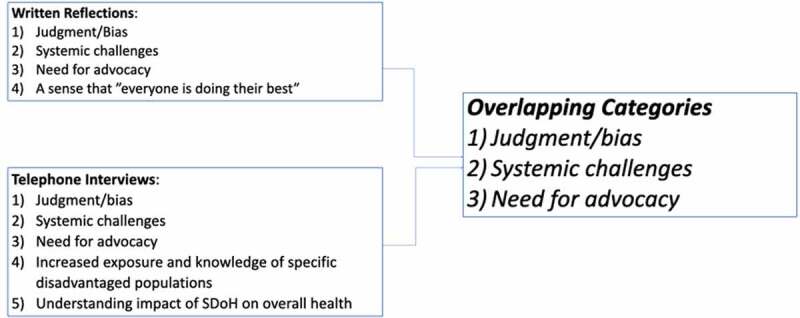
Categories Generated.

Based on our analysis, the *written reflections* revealed four saturating categories: 1) judgment/bias, 2) systemic challenges, 3) need for advocacy and 4) the sense that everyone is doing their best. As with the written reflections, the *telephone interviews* resulted in the three similar categories of judgment/bias, systemic challenges, and advocacy saturating, along with the additional two categories: 1) exposures to new populations and locations; and increased knowledge of specific disadvantaged populations; and 2) understanding the impact of SDoH on overall health (Figure 1). Table 1 includes direct quotes taken from written reflections and interviews, which highlight each of the saturated categories as listed below.

**Table 1.** Findings: Saturated Categories from Reflections and Interviews

Categories	Direct quotations from Interviews	Direct quotations from Reflections
Judgment/Bias	‘I did find myself surprised sometimes when all we had was a well baby visit and it was actually a well baby … So I would walk into half these appointments going, “Okay, what’s the catch? What’s the catch?” But sometimes it actually just was a cute, well baby.’ (Interview 2) ‘I think there’s a lot of biases that come out. I, for example, in that teaching session that I had, I went in with this bias that it was going to be hard, because I was coming in with a different colour of skin and a different background and a different language … But all of those things also come from the other side. I think they probably had feelings of, it could be either direction. Like, “Oh she’s going to tell us what to do or think she’s better.” It would also be like, “I’m really nervous, she’s going to judge us.”’ (Interview 6) ‘But there’s so many different things that I think from a bias standpoint that we bring in, that you have to actively work to overcome, which is too bad. I wish, I’m hopeful for one day that that’s not the case. I think there’s a lot of biases that we bring in, probably that they bring in too. But I think as a healthcare provider, it’s your job to be the one who actively takes that role of overcoming this and helping lay the groundwork for them [the patient] to feel comfortable.’ (Interview 6) ‘But I think that experience has just kind of given you that reminder, even when I am frustrated or finding it difficult, to really think about where this patient is at and to kind of try and recognize if there’s a bias for some reason or I’m judging somebody and to take that out of the equation to make sure that they’re getting the care that they should be getting, if that makes sense.’ (Interview 5)	‘I thought that there was no way this mother could cope with life, especially that she was a single mother who did not speak English. I felt sorry for this family and desperate at the same time because I thought that no service or support would help them to manage their life.’ (Reflection 22)
Systemic challenges	‘Yeah, just coming back to X community, the healthcare system is not meeting the needs of that community. I think it’s horribly under-resourced … It’s shocking that there’s two physicians servicing that large population with … high medical needs. I think, absolutely, the healthcare system’s failing on reserves. It’s not even a money problem, sometimes. A lot of it is recruiting.’ (Interview 1) ‘Through this rotation and through getting to know some families with many challenges, I am reminded how difficult it is to access services and how systems are not necessarily set up for people that they are meant to “help”. The bureaucracy and barriers that exist are a tremendously large and often insurmountable hurdle for patients and their families to overcome.’ (Interview 2) ‘I don’t think we do enough to help vulnerable populations, by a long shot. And I think for certain populations, we go out of our way to the point where I think it is harmful. I do think that, I remember having a super cynical thought; we had an Indigenous Awareness Week, a few months ago at the hospital. And as part of that, I remember someone coming around, “Oh here’s a cupcake” … and I was like, “Oh here’s like a bag of candy” or something like, we’re just celebrating and doing this. And I’m like, I remember cynically saying, “Is that all we could do?” Here’s a bag of candy for these patients. That’s not going to change what’s going on in terms of just trying to reverse everything that’s happening ever. Right. You know what I mean? I remember thinking, “What a weird gesture.” I just couldn’t process it. It’s just like, it’s nice, but it’s not enough.’ (Interview 10) ‘But I think for her, she made me view the system a little bit differently. The phrase that she used would be: the system offers “good enough care” as opposed to good care. So as long as it’s better than the situation they were in before, then that’s already a step up. Is that enough for our kids? Are we failing our kids by only saying like “Well it’s good enough,” as opposed to it being actually good? I don’t know the details about the system enough, but there are those kids where you’re like, why are they still … they’ve been here for two years, their parents don’t visit, how are they still the guardians? You have those situations where you feel there’s a bit of a disconnect between what maybe personally you feel should be happening with this child versus what the system is doing for the child.’ (Interview 4)	‘The system is really stacked against marginalized populations. Systemic racism, child protection services that are unable to protect every child, unstable foster homes, kinship arrangements that seem like a compromise to make the child a little bit “safer”. I think my previous assumption was that once you activate the system, good people would come in and make it right, but I now more clearly see that that isn’t always the case.’ (Reflection 19)
Need for advocacy	‘And then the day-to-day things that you can do as well are making sure that you advocate for your patients. So if they need services, that you don’t think they’re getting, then we have a powerful position to write letters on their behalf, talk to schools on their behalf, attend meetings on their behalf and work in a collaborative environment so that everyone’s on the same page.’ (Interview 9) ‘I think the thing that I really took away is that you can really make a big difference with advocacy, but then there were sometimes where no matter how much you advocated, the system wasn’t set up to be responsive to that, so you couldn’t necessarily make the changes that you want. But it’s still, unless you advocate, nothing is going to change.’ (Interview 4)	‘I realized that the interaction with this patient, or with any patient for that matter is not about my goals but about listening to the patient and their needs.’ (Reflection 16) ‘We must advocate for our patients by preventing trauma, treating trauma, ensuring food security, ensuring stable housing, etc. We need to actively work towards identifying those at risk and building resiliency in these patients. For me, this is my goal and commitment to my patients going forward.’ (Reflection 27)
Everyone is doing their best	‘I think there comes a point where you have to accept you’ve done everything you can and you set up follow-up and you do all these things to try and make the environment as safe as possible. But part of it, that’s where social peds comes in. Just because things are going to be different and not the way you want them to be, or expect them to be in every place. And so part of that is just the nature of this whole experience. So managing that as best as you possibly can.’ (Interview 6)	‘Though she had issues of her own, she was making strong efforts to prioritize the health of her child … She was providing consistent care for her child and, based on the child-mother interactions observed, they had a strong and loving bond.’ (Reflection 14)
Increased exposure and knowledge of specific populations and locations	‘I think a lot of us in the hospital, we’re so lucky to have all these people to help us figuring out resources, but not everyone is so privileged. And probably in those smaller communities, they might not have … we’re kind of right in the centre. Like we’re in a big city where if we needed things, it would probably be much easier than if you’re remote. I do think that part of this rotation, it’s really helpful to go to one of the smaller communities. Even just doing a trip to, I think they’re day trips to Cold Lake, but I really liked getting that time in High Level to realize these outreach clinics are kind of what a lot of people are depending on for their care.’ (Interview 4) ‘And seeing so many traumatized kids and so many kids in foster care coming in through that clinic as a proportion of the clinic was also really eye opening.’ (Interview 2) ‘Yeah, by giving us access to those clinics that we would never have seen. And then by being in those contexts, we get access to patients that we may never have seen in other clinics as well.’ (Interview 4) ‘And I know sometimes in clinics we skip over the, do they have coverage for medications, or for some First Nations kids, do they have treaty status? Are they covered by NIHB? But it makes such a huge difference if you can get them covered for Tylenol, Advil, get them put on an antibiotic that’s appropriate, that’s also covered. Get them put on a puffer that’s actually covered. So, that experience really made me think of that and go that extra step for my patients on CTUs (clinical teaching units), especially. Today, I learned how to write a prescription for Pampers. Who knew? But you can apparently get that covered as well.’ (Interview 2) “As I transition into practice, it’s more about being able to navigate resources and what’s out there that you can offer to these families and hopefully helping them improve their current conditions. And I think now the other thing I’ve learned is when you’re looking for resources, not just looking at the one that you think would be good for them, but having backup options. And saying, ‘if there’s three that they’re eligible for, I’m actually going to refer them to all three and whichever one comes first. And then you can cancel the other referrals.’ (Interview 7)	
Increased understanding of SDoH on overall health	‘We talk about the living situation of patients in hospital a lot, and in pediatric clinic, but I think going to see it in person and getting to, not that it wasn’t real, but it makes it more … You can see the true extremes of how people live. So I think for me it was eye opening in the sense that I was like, how do people actually live like this, and have a sustainable life? How does this reflect other aspects of their life if they can’t even really take care of their home.’ (Interview 4) ‘Just the nature of it, unfortunately, the result of lots of systemic racism and drug abuse and multi-generational abuse and trauma that really does lead to kids having all of these blocks ahead of them that they have to overcome before they can even look at furthering some of their basic needs and their basic health.’ (Interview 8) ‘I think in general, anytime you work with anyone who has any barriers or notices any of the social determinants of health impacting their care, it reinforces that there are a lot of populations within Alberta that do have a lot of disparities and there is a kind of hierarchy. So we just have to be very mindful of ensuring that we can provide as equitable care as possible for our patients.’ (Interview 9)	‘You can read about the social determinants of health and see the list of ten things, but until you talk to a family and hear that they’re going back into hospital because they couldn’t afford $73.11, just really imprinted differently on your brain. So, taking that forward is about looking at each individual family, not just as a problem that I can look up on UpToDate, it’s a whole family is something that you need to find a treatment plan for.’ (Reflection 2)

### Category 1: Judgment/Bias

Residents expressed experiencing direct bias towards their patients and their families in a variety of situations. Some residents noted biases wherein they assumed patients would be doing poorly based on the contexts in which they were seeing them. Interestingly, a number of implicit biases were evident in the residents’ reflections (Table 1). These were directed towards patients, their family members or their environments. The residents also described implicit biases regarding certain families’ ability to cope with the challenges of having a sick child.

In addition to realizing biases towards certain patients, there were circumstances wherein residents felt biases directed towards themselves. This was expressed (quotes in Table 1) with reference to both clinical situations and educational events that residents attended. Most frequently residents commented on biases they felt in the form of racism. They also identified the importance of being aware of, and challenging biases, most notably their own and those of their colleagues. Furthermore, some acknowledged the need to actively prevent these biases from affecting their work and patient care.

### Category 2: Systemic challenges

Within the written reflections and interviews, residents frequently commented on systemic limitations that impacted their patients. In general, they identified access to care as a major barrier, in addition to a sense that the healthcare system was failing their patients. Limitations in certain geographical locations, such as rural and First Nations reserve communities, were most often identified as having increased barriers to care. Even when geographical location is not a barrier, many residents identified that certain disadvantaged populations experienced challenges in accessing services due to various logistical challenges, such as not having personal identification, fixed addresses or even a telephone for communication of appointments.

Experiencing a shortage of resources also arose as a systemic challenge that residents faced in caring for their patients. Specifically, many residents noted that resources for mental health and help for substance users were particularly challenging to access. Many residents felt as though the healthcare system was failing certain patients especially those in foster care. The feeling that the system was failing these children, came up frequently.They also felt that a lack of focus in preventative health led to more disparties in , the general system as a whole.

### Category 3: Need for advocacy

Residents reported numerous experiences wherein they witnessed advocacy or expressed increased recognition for the need to advocate for individual patients. Similarly, residents reflected on the importance of advocating for patients in such a way that aligns with the patient and family’s goals through patient-centered care. What’s more, some residents expressed that their experiences throughout their Social Pediatrics rotation sparked increased desire to advocate for patients in their future career.

### Category 4: Everyone is doing their best

Residents frequently highlighted that throughout their Social Pediatrics rotations they were exposed to patients, families and health-care providers who, despite many challenges, were doing their best to provide care. Many people expressed parents’ efforts as an asset to their patients’ overall health, often despite their own challenges. In addition to this, residents described challenges in providing care in clinics or contexts wherein patients frequently have more complex social circumstances. They described that sometimes it is not realistic to make perfect management plans that are ‘by the book,’ but that some degree of compromise is necessary.

### Category 5: Increased exposure and knowledge of specific populations and locations

Our study participants reported ‘eye opening’ exposures to new communities during their rotation, specifically reserve communities, and outreach clinics in rural centres as far as 800 km away from Edmonton. Given that Edmonton has a large encatchment area, the rotation gave residents a chance to see the communities that many of their patients live in. Other residents commented (Table 1) on the unique experiences that were part of the Social Pediatrics rotation including opportunities for intentional exposure to specific population groups. These included new immigrants to Canada, children in foster care, high-risk youth, and adolescent mothers.

Many residents identified that these exposures to specific marginalized populations and remote locations developed their approach to history-taking, specifically targeting social determinants of health in addition to more traditional review of systems. What’s more, residents commented that their experiences gave them increased confidence to have what are often perceived as difficult conversations regarding more sensitive subjects, such as finances, trauma and abuse. They also displayed awareness of asking questions to ensure the management plan is suitable for the patient and their family.

Residents also identified that their rotation gave them increased knowledge of community resources and how to access them. They emphasized the importance of not only being aware of resources, but also learning how to access them and support patients in doing so.

### Category 6: Increased understanding of SDoH on overall health

Residents frequently commented on an expanded understanding of SDoH and how these impacted the care of their patients. Many residents discussed the importance of the environment in which their patients shaped their care, including family structure, living circumstances and finances. In addition to the aforementioned factors, exposure to adverse childhood experiences (ACEs) in the children or their family members was commented upon as a source of adversity (Table 1). The residents expressed that although they were aware of the importance of living environments on health prior to their Social Pediatrics rotation, having a firsthand look into the lives of their patients reiterated this and made it more meaningful. Perhaps as equally as important as an increased awareness of SDoH, residents expressed how these must be accounted for in providing patient-centered care.

## Discussion

This is the first study to our knowledge to explore learning around the social determinants of health, its influence on access to care, and the health-care team providing that care through reflective writing. The four saturating categories that were generated from the *written reflections* were judgment and bias, systemic challenges, need for advocacy, and a sense that everyone is doing their best. The *telephone interviews* revealed the first three categories (bias, systemic challenges, advocacy) with overlapping saturation, along with two new categories: an increased exposure and knowledge of disadvantaged populations, and understanding the impact of SDoH on overall health.

### Generated theories for teaching SDoH in residency

The three *overlapping categories* from the written reflections and interviews (bias, systemic challenges, advocacy) are key concepts one must wrestle with in the pursuit of equitable healthcare, but these topics are not explicitly taught during medical school or residency. The interviews revealed that when residents approached clinic encounters using a social pediatrics lens, they were able to understand the true impact SDoH play on the overall health of a child. The residents acknowledged the importance of checking their own biases and judgment before interacting and assessing patients. Many conscious and unconscious biases in medicine are not just from ones carried over from past life experiences and belief systems, but also ones the learner has unconsciously picked up during their training from fellow trainees and staff physicians whom they may respect. This forms part of a hidden curriculum steeped in the inherent biases of western (some would say colonialist) medical system that affects how we approach and treat people of different demographics and cultures. More and more cases of racial biases in Canada’s healthcare system are now being highlighted in the mainstream media. In 2008, Brian Sinclair, an indigenous man, died in a Winnipeg emergency department while waiting 34 hours for care. Although an inquest and final report completed in 2014 concluded that his death was preventable, Barry Lavallee, a professor at the University of Manitoba and a member of the Brian Sinclair Working group states, ‘The recommendations from the inquest itself did not and will not interrogate racism.’ The working group’s interim report, Out of Sight, concludes Sinclair’s death was a result of racism in the health-care system [14]. A more recent case in the news in 2020 was that of Joyce Echaquan, an indigenous woman in Quebec, who filmed hospital staff taunting her. She died later the same day she filmed the video [15]. A systematic review looking at implicit bias in health-care professionals indicated that implicit bias leads to disparities in healthcare [16].

Residents also came to appreciate the systemic challenges in our healthcare system and the importance of advocacy by working with health-care practitioners who delivered care in locations that do not have the physical or human resources as a tertiary care centre does. On the Government of Canada website for Canada’s Health-Care System [17], fiscal restraint is listed as a challenge for how services are delivered. It is well known that there is a high cost in delivering healthcare to our remote communities and the vast distances that families need to travel in order to access healthcare. The lack of or limited access to quality healthcare is not truly appreciated until one works outside urban center (where most training programs are situated). During the rotation, residents were able to travel to outreach clinics in Northern Alberta (including ones that they needed to fly to) and on First Nations reserves. Similarly, the learners were able to experience firsthand the barriers facing homeless youth when working in a clinic situated in a youth shelter, and the struggles individuals face when they present to a clinic in the inner city. Through these placements they witnessed the importance of addressing health inequities by advocating for those who live in underserved and resource-poor settings. By working with preceptors who worked in these settings, they learned how to advocate for services, supplies, and medications in different ways than they were traditionally used to, i.e., regular healthcare coverage.

The additional categories that were saturated from the interviews confirmed that the main objectives of the rotation were met: the learner was exposed to new populations that they had little to no previous experience with and saw first hand how SDoH impacted the patient-centered care they were able to deliver. This is a crucial finding to highlight. It is well known that trainees most frequently will settle to practice in the community or a community similar to ones they have been already exposed to in their life and/or during their training. If they have not worked with or been exposed to certain populations such a Indigenous families on a reserve, refugee families being seen in a resource poor clinic etc., they do not incorporate or consider these settings into their future careers. A questionnaire-based study by Larkins et al. [18] showed recruiting trainees from underserved groups (lower socio-economic, non-urban status) led to a higher proportion of students who intended to practice in non-rural settings. The University of British Columbia (UBC) has successfully placed increasing numbers of graduated physicians in more northern and remote communities by expanding and distributing medical education. As a result, more undergraduate and graduate trainees were placed in more rural settings. The ‘evidence shows that as students gain exposure to the people and places, as well as the rewards of practice, they are more likely to set up shop there’ [19]. This is the reasoning behind why training programs are advocating for more funding directed to train rural physicians. During their rotation, the residents witnessed the importance of SDoH in the delivery of healthcare and recognized that resources are disproportionately allocated to urban and hospital settings.

### Limitations

As with any research study, we identified some limitations to our study. Firstly, our data was collected from a single institution. In future, it may be beneficial to expand similar studies to other pediatrics residency programs and other specialties across the country. Secondly, given that there are approximately 10 residents completing the Social Pediatrics rotation per year, there is limited availability of data to analyze at any given time. This similarly may be addressed by expanding the study to other residency programs. Finally, the principal investigator also leads the Social Pediatrics rotation; the possibility of bias was mitigated by having clinicians and researchers code the data and ensure reflexivity.

## Conclusion

During the four-week Social Pediatrics rotation, residents work with underserved populations in their home communities and gain an understanding of the structural and social barriers families face that impact the wellbeing and health-care needs of their children. We believe this is a new and innovative rotation as showcased by the resulting work produced by the residents in their final reflective assignment. The assignments have been rich in content and common categories were generated. These categories can be used not only to reinforce the importance of a social pediatric rotation during pediatric residency training, but one can extrapolate these findings to highlight the importance of social medicine teachings earlier in training and across all disciplines. Advocacy is something that cannot be taught in a classroom setting, one can model behavior and have mentors. Physicians learn and are motivated to advocate for their patients once they start clinical work and notice disparities in care. The learners who participated in this study learned from the health-care professionals they worked with. The staff helped highlight the social determinants of health, which needed to be addressed but also helped to teach the learners about the structural determinants of health, which require us as a society as a whole, to address. Only once we do so, can we then start to address the inequities that exist in our healthcare system in all domains.
